# Early onset colorectal cancer: Challenges across the cancer care continuum

**DOI:** 10.1016/j.amsu.2022.104453

**Published:** 2022-08-22

**Authors:** Adhari AlZaabi, Amna AlHarrasi, Atika AlMusalami, Nawal AlMahyijari, Khalid Al Hinai, Humaid ALAdawi, Humaid O. Al-Shamsi

**Affiliations:** a Sultan Qaboos University, Sultanate of Oman; b Sultan Qaboos Comprehensive Cancer Care and Research Center, Sultanate of Oman; c Royal Hospital, Sultanate of Oman; d Oman Medical Specialty Board Council, Sultanate of Oman; e Sultan Qaboos University Hospital, Sultanate of Oman; f Medical Oncology Department, Alzahra Hospital Dubai, Dubai, United Arab Emirates; g Department of Medicine, University of Sharjah, Sharjah, United Arab Emirates; h Emirates Oncology Society, Dubai, United Arab Emirates

**Keywords:** Early onset colorectal cancer, Screening, Survivorship, Palliative care, Adolescence and young adult, Prevention

## Abstract

Early Onset Colorectal cancer (EOCRC) incidence is increasing at an alarming pace. An increase of 90% in colon cancer and 124% in rectal cancer is expected by 2030. Patients with EOCRC are not receiving additional attention compared to older patients despite having a unique molecular pattern, majority of cases are sporadic, and related short- and long-term treatment and disease complications. The current management and screening guidelines have been constructed from studies on late onset CRC. Plethora of studies are ongoing to understand this disease entity in order to construct a tailored prevention, detection and management plans. While waiting for a better understanding of the disease, efforts should be directed toward improving the quality of care across the cancer continuum. Here we aim to address the challenges faced by EOCRC patients across the cancer continuum. This will facilitate directing future efforts and research toward construction of a personalized and precise guidelines.

## Highlights

• Studies showed that Ealy onset colorectal cancer is caused by an accumulative risk exposure.
• Indirect cost of premature death and reduced productivity due to EOCRC exceeds direct costs.
• Patients, system and physicians related diagnosis delays should be improved.
• Young cancer patients have unique survivorship concerns different from old patients.

## Graphical abstract

**Figure:**
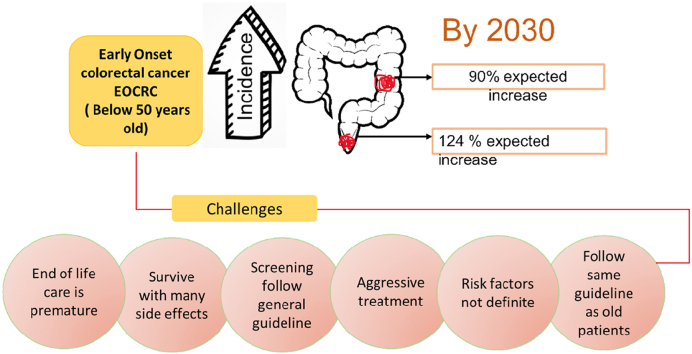

## 1 Introduction

### 1.1 Cancer in adolescents and young adults

“You have Cancer” hearing these three words at the age of 15–40 years old is very distressing. Yearly, about 90,000 young patients are diagnosed with cancer in the United states which constitutes about 12% of all diagnosed cancer [1]. This age group usually suffers from unique obstacles compared to those who are above 40 years old. People at this age are usually busy building their career, families and discovering opportunities. Mentally, psychologically and socially, they are not ready to accept a sudden interruption of life and entering such a life trajectory. Many distressing issues arise related to disruption to work, financial concerns, increased dependence on others when they are ready to be independent, and existential distress related to a premature confrontation with mortality and a sense of lack of life completion [[2], [3], [4]]. Despite their unique challenges and needs, they have not received extra attention and historically they have been considered as understudied research population.

### 1.2 Early onset CRC (EOCRC)

Colorectal cancer (CRC) is considered a serious health issue with a significant burden of the disease worldwide. Despite advances in colorectal cancer screening strategies, diagnostic tools and therapeutic options, it still remains the 3rd most common cause of cancer overall with a high mortality rate relative to other forms of malignancies [5]. Since 1990, researchers noticed an increase in incidence of colorectal cancer (CRC) among those who are below 50 years old which is labeled as Early onset CRC (EOCRC). An increase of 17% and 75% in colon and rectal cancer respectively was reported between 1973 and 1999 among those 20–40 years old [6]. Overall, a yearly increase of 1.5% in males and 1.6% in females for colon cancer and about 3.5% in males and 2.6% in females for rectal cancer has been reported [7]. It is that by 2030, the incidence will increase by 90% and 124% for colon and rectal cancer respectively [8]. It was thought that it is mainly due to the successful uptake of screening programs among those above 50 years old. Later it was confirmed that it is a real increase which requires investigations [9]. Globally, many studies are directed toward deciphering the causes of such increase and to understand the molecular signature and prognosis of CRC in this age group.

**Fig. 1:**
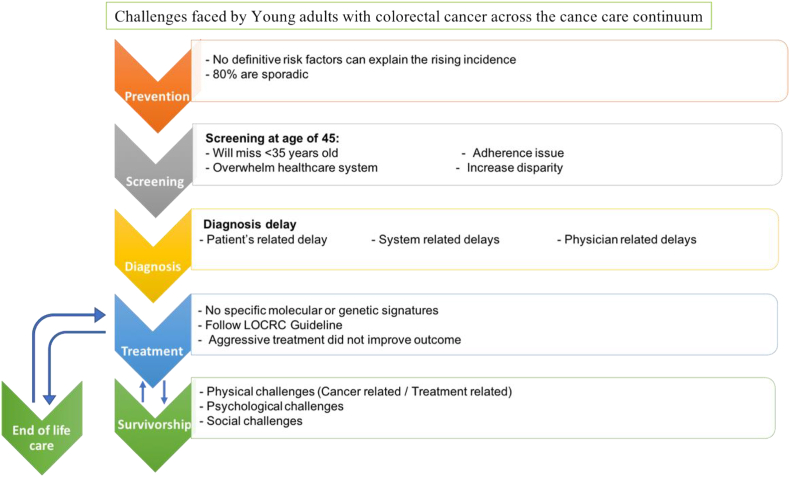
Challenges faced by Young adult with colorectal cancer across the cancer care continuum.

Despite the unique needs and obstacles of EOCRC patients, they follow same guidelines that were constructed from studies on late onset CRC (LOCRC) patients. Therefore, the current guidelines of the cancer continuum are proven not to be applicable to these patients [10]. In a qualitative focused group study done by a team from American National Cancer Institute, patients with early onset cancer 9 < 50 years old) reported concerns across all the cancer care continuum and they addressed the need for a tailored continuum that considers their needs and improve their journey. Here we aim to highlight the challenges listed in literatures that are faced by this unique population across the cancer care continuum starting from prevention, detection, diagnosis, treatment and survivorship and end of life care (Fig. 1).

## 2 Prevention and risk reduction

Colorectal cancer is largely a preventable disease through avoidance of modifiable risk factors and early detection. It was thought that EOCRC is strongly related to hereditary syndromes. Epidemiological studies showed that <5% of EOCRC is attributed to inherited familial cancer syndromes. About 5% are familial and less than 1% are caused by inflammatory bowel diseases. The vast majority of EOCRC are sporadic [11]. It is believed that the development of sporadic colorectal cancer is multifactorial. Modifiable risk factors such as physical inactivity, sedentary behaviour, and excessive caloric intake have been largely blamed [12,13]. All of these factors collectively lead to obesity as a sequence of what is called energy imbalance which has been proven to be a significant risk factor for early-onset colorectal cancer especially in females [13]. Therefore, it is thought that the recent steady global increase in EOCRC incidence is due to the widespread implementation of inactive lifestyles and unhealthy eating habits [14]. Other attributing factors are the increase incidence of obesity and diabetes mellitus (DM) and smoking as well. DM was found to be associated with 20–38% increase risk of CRC [15,16]. The association was found to be highly dependent on the duration of DM where a significant association reported in patients with DM for ≥ 10 years while no significant association in those with DM for <10 years [17] Fig. 1.

**Fig. 2:**
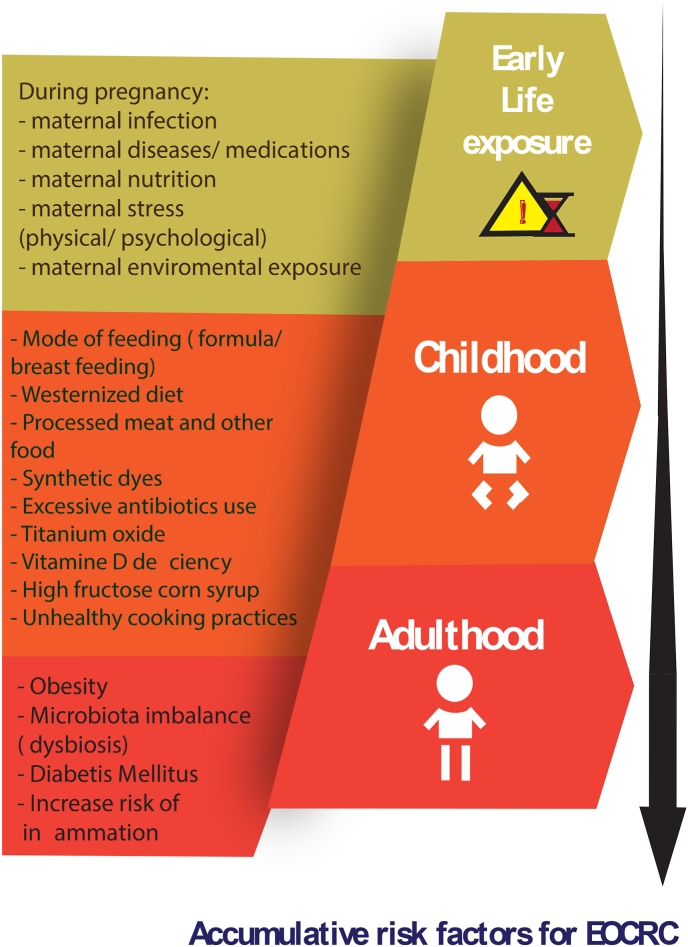
The reported accumulative risk factors for EOCRC.

Looking closely at this claim, smoking, sedentary lifestyle and DM incidence have increased among all age groups not only young people. Therefore, it can not fully explain the increase incidence of CRC among those younger than 40 years old. Furthermore, Chen et al. reported that EOCRC patients have low rate of obesity and overweight compared to LOCRC patients [18]. Also, there are repeated observations of clinicians that EOCRC patients are generally active, fit with no comorbidities [19,20]. Since these are all observational studies, causation and risk factors association can not be concluded precisely. In the other side, a metanalysis has proven that physical activity was associated with significant reduction in the risk for colon cancer by approximately 30% [21]. It is worth mentioning here that studies are still going to evaluate the associations of these factors with EOCRC. Other factors that are currently under investigations as well are the association of excessive consumption of processed meat, sugary drinks, excessive antibiotics use either in childhood or even in utero, early life exposures and other proposed factors that need to be critically evaluated to understand their association with increase incidence of CRC among the young. It is thought that it is an accumulative risk that increases the risk of CRC at adulthood (Fig. 2). Starting from in-utero exposure, mode of delivery, mode of feeding at early life till all environmental exposure in childhood. These factors together thought to collectively disturb the colon microbiota which ultimately lead to increase risk of inflammation and malignant transformation [22].

## 3 Screening and diagnosis

The stage of CRC at time of diagnosis and the early initiation of treatment is what dictates the prognosis and survival rates. For instances, patients with a lower stage (Duke A) have a 93% 3-year survival rate compared to a significantly lower (16%) rate for those with more advanced tumors [23]. A greater proportion of EOCRC patients are diagnosed with advanced-stage compared to LOCRC. Several factors might explain such difference, more importantly is the delay in diagnosis which warrant that physicians and patients should seriously consider alarming GI symptoms, dig into family history and request the needed evaluation workup in suspicious cases [24]. The two widely used means to detect CRC at an early stage and thus reduce its incidence and disease burden are screening programs for asymptomatic patients and early diagnosis in symptomatic patients.

### 3.1 Screening for EOCRC

The aim of screening is to identify and remove pre-malignant lesions and detect early stage disease in asymptomatic people to improve outcomes. This has been evident in the 45% reduction of CRC incidence and the fall of CRC-related mortality by more than half [25]. CRC Screening Guidelines do exist worldwide, but differ regionally according to the burden of the CRC in the country. Examples of the most preferred screening methods for those >50 years old are colonoscopy every 10 years, flexible sigmoidoscopy every 5 years, yearly guaiac-based faecal occult blood test, faecal immunochemical test every other year or multitargeted stool DNA every one to three years [26].

Recently, and as a response to the rapid increase in the incidence of EOCRC, the US Preventive Services Task Force (USPSTF) has made a significant change in the CRC screening guideline [27]. The new guideline is to initiate CRC screening for average risk patients at the age of 45 years instead of 50 years. This change is a translation of collective evidences concluded from a systematic review [28] an epidemiology data [29] and a modeling study [30]. Some other countries even lowered the screening of CRC to start at age of 40 taking into consideration their cancer data [31]. Several concerns are raised based on these new guideline. First, the net benefit will be as expected only if the adherence of the targeted adults is 100% which in reality is not the case. Knowing that the adherence to CRC screening program has been an issue for a long time. Despite all the efforts taken to encourage eligible adult, studies showed that the CRC screening uptake in the USA remains suboptimal [32]. In the US, one study showed that the adherence rate over a 10 -year interval was 64.3%, which falls short of the recommended target of 80% [33]. This means that screening will miss at least third of the target population. In addition, among all participants in the USPSTF screening program, who initially showed interest, become gradually non-adherent with time especially with FIT or gFOBT tests that require to be repeated on annual basis and are often negative. The rates in one study have been shown to reduce from 38% at year 1–18% at year 2–12% at year 3 [34]. Therefore, the adherence of younger people can not be guaranteed because the net benefit might not be realized knowing that majority of cases are sporadic with no prior risk factors. Second, stratifying the incidence by age shows that almost half of EOCRC cases do occur in those who are younger 45 years which will be missing in the new guideline. Third, it is thought that reducing age of screening is expected to improve the outcome of those who have good access to screening program and subsequently will increase the health disparity gap for those with minimal access. Several observational studies have reported an already existing racial/ethnical health disparity in the detection, diagnosis and prognosis of EOCRC patients [35,36]. The new guideline is suspected to increase this disparity further. Fourth, the new guideline will add about 20 million people as eligible population which means about 10.7 million extra colonoscopies in addition to the currently conducted scopes. This will worsley impact the current suboptimum CRC screening uptake due to unavailability of services [37]. Therefore, instead of considering age as the only determinant factor for screening initiation, a personalized approach would be more beneficial through adoption of risk assessment tools which would determine subsequent risk modification strategies such as screening or prophylactic intervention for high risk people.

### 3.2 Diagnosis of EOCRC

**Fig. 3:**
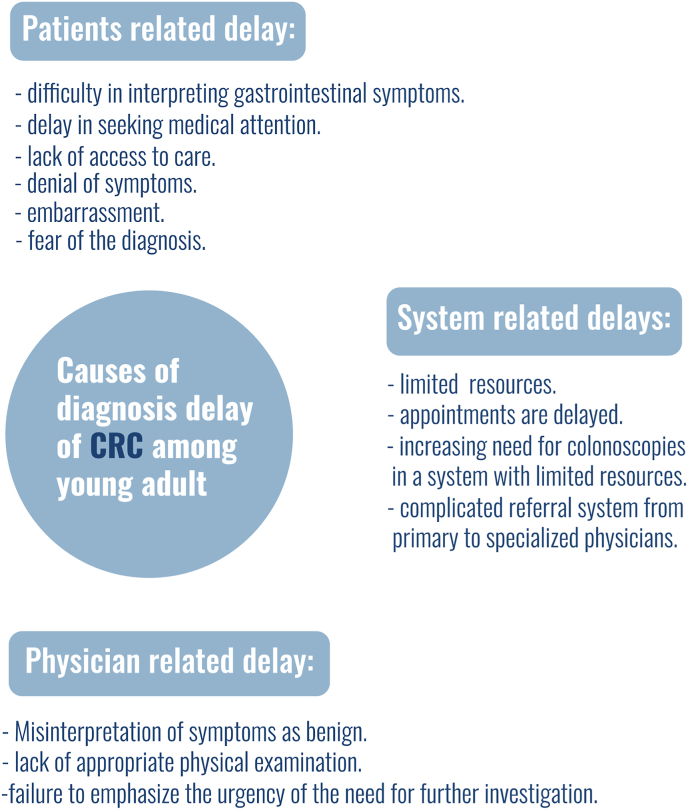
Different levels of diagnosis delay of colorectal cancer among young Adults.

Young patients with CRC are usually identified based on a thorough evaluation of suspicious symptoms and signs [18]. Diagnostic delays have been cited as a major challenge facing EOCRC patients [38]. Diagnosis of CRC in young symptomatic patients is usually not a straigt forword task. The complexity lies in the fact that this often involves multiple steps each leading to delays before a formal diagnosis is made [39] which can be divided into patients related delays, system related dalays and physician related delays Fig. 3. The first cause of delay in the chain of delays is patients related delay which is mainly due to difficulty in interpreting gastrointestinal symptoms. A meta-analysis published in 2008 looked at data from 15 studies with a total of 19443 patients found that symptoms are poor identifiers of CRC with a sensitivity ranging between 5 and 64% [40]. The poor correlation between symptoms and CRC is thought to be because symptoms are often nonspecific and mostly caused by non-malignant conditions [41]. As a result patients fail to recognise their symptoms as concerning or serious, which leads to a delay in seeking medical attention [42].In one study, it has been estimated that 18% of patients with rectal bleeding and 37% of patients with a change in bowel habit delay their symptoms by about 1 month [43]. In average, the average time from symptoms to seeking medical advice for symptomatic young adult is approximately 6 months [44]. Other reported reasons of patient related delays are lack of access to care, denial of symptoms, embarrassment and fear of the diagnosis [44].

Another challenge in diagnosing CRC is physician-related and this happens by two main ways. Incorrect diagnosis is the first with a rate of 31–34% and in those patients it leads to a delay in diagnosis of CRC of about 200 days [42]. In this case, misdiagnosis occurs because physicians attribute their patients’ symptoms to benign conditions and thus fail to perform further investigations. The second way in which this happens is the lack of appropriate physical examination. Langenbach et al. reported that a rectal examination is not performed in 75% of patients with rectal cancer [39]. Even when a malignant process is suspected failure to emphasize the urgency of the need for further investigation when referring those patients can lead to unnecessary delays before a formal diagnosis is made. For instance, a delay of about 92 days has been noted because of this in patients with a positive occult blood test [45]. Another challenge lies in the increasing need for colonoscopies in a system with limited resources [42].

**Fig. 4:**
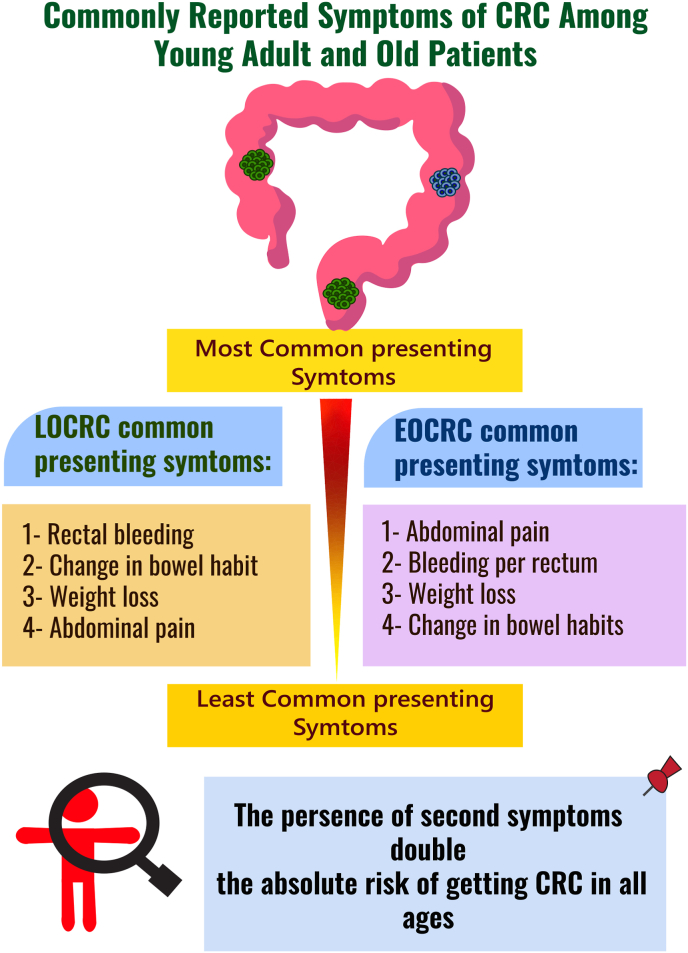
The most reported presenting symptoms among young and old colorectal patients.

Risk assessment tool or an EOCRC symptoms index might be helpful to classify patients into high risk who need immediate intensive work-up and low risk who need reassurance and follow up. A symptom index has been constructed earlier by Goff [46] for ovarian cancer and was validated in different countries [47]. Knowing that the commonly reported symptoms of CRC among young population according to a systematic review of a total of 6425 patients are abdominal pain (55%), bleeding per rectum (46%), weight loss (35%) and a change in bowel habits (32%) [9,48]. Rectal bleeding is a common symptoms that should never be ignored. The presence of 2nd symptoms double the absolute risk of getting CRC in all Ages (Fig. 4) [49]. There are previous works to construct similar indixes. For example, Adelestein [50] has constructed a self administered questionnaire for CRC prediction which was found to be reliable. Another predictive tool was developed by Freedman [51] which was tested and validated on those above 50 years old. Freedman questionnaire included symptoms and known risk factors and found to be of good validity and provide a powerful prediction [51]. How effective they are in the detecting EOCRC or in improving screening is yet to be studies. Therefore, symptoms index for EOCRC along with increasing awareness of the public with an emphasis on the frequency and duration of every symptom could influence young adults to seek medical advice earlier, thus minimising diagnostic delays and reducing mortality related to late diagnoses. Shifting the EOCRC diagnosis into earlier stages could be translated into saving lives and reducing the direct and indirect costs of cancer care. Siegel.et al. reported that almost 40% of all CRC deaths can be prevented if patients were diagnosed at stage III instead of stage IV [52].

### 3.3 Economic burden of EOCRC

Unfortunately, despite all above findings with the availability of advanced treatment protocols, the reported 5 years survival rate is only 14% for the EOCRC [52] contributing to premature death. The cost of cancer care in general is very expensive considering both the direct and the indirect costs adding to it the years of potential life lost due to premature cancer-related mortality. The low survival rate of CRC patients in general and the EOCRC patients specifically and the advanced stage at presentation contribute to the loss of the productivity years of these patients and the extra economic burden. In 2018, about 16.63 billion united state dollars (USD) was estimated to be the national direct medical expenditures on cancer care in the United States [53]. In fact, the indirect cost due to premature death and reduced productivity are reported to be exceeding the direct medical costs of direct cancer treatment [54]. For example, in 2008 in Ireland, the estimated cost for the premature death of colorectal cancer was USD 262,110 per death compared to USD 45,752 for calculated cost of direct cancer care including diagnosis, treatment and follow-up [55]. The across Europe study in 2015 reported The economic burden of colorectal cancer across Europe to be about €19·1 billion where 60% (€11·6 billion)of it is non-health-care cost [56]. In addition, absenteeism from work and reduced work productivity caused be the disease or its treatment should never ignored [57]. A recent systematic review by Khalili.et al., 2020, concluded that establishing a screening strategies tailored to the specific population is better than no-screening strategy [58]. It has been proven that it is cost-effective to prevent or early detect precancerous lesions than to attempt treating advanced CRC with major surgery and expensive chemotherapy.

## 4 Management of EOCRC

### 4.1 Hereditary vs non-hereditary

International CRC screening guidelines are based only on age and family history despite the fact that almost 70–75% of CRC cases are sporadic with no family history of the disease especially in those below 50 years old. The remaining 25–30% of CRC cases are either inherited or familial cases. Inherited CRC represents only 10% of all diagnosed CRCs and is considered in patients who present with either CRC at younger age, present with multiple primary tumors or if there is a familial aggregation cancer [59]. The inherited syndromes are divided into two categories, polyp associated syndromes such as Familial Adenomatous Polyposis (FAP), MUTYH-Associated Polyposis (MAP) and hamartomatous polyposis syndromes and non-polyp associated syndromes such as Lynch syndrome (HNPCC). Having a family member with CRC due to any of these syndromes necessitates early screening and sometimes prophylactic procedures for people in the family who are at risk to reduce their chance of developing CRC. The remaining CRC cases are familial which do not follow well understood pattern were patients report positive family history of CRC that doesn't follow a clear pattern consistent with a known inherited syndrome. Having a family member with familial CRC below the age of 55, increases the risk of developing CRC by 1.7 folds for other members. The prevalence range of hereditary syndrome in EOCRC was reported as 9–26% with an average of ≈13% [59]. The remaining vast majority of cases are sporadic.

### 4.2 Molecular signatures of EOCRC

It was thought that sporadic EOCRC represent a distinct entity from sporadic LOCRC at the genetic, molecular and clinical level. Recently, there are studies that reported a distinctive molecular difference [60] while other studies found no unique differences neither at the molecular level nor in the gene of the CRC driver pathways [61,62]. The only reported difference was in the level of *BRAF V600E* and *APC* variants which were found relatively low in EOCRC, but no significant association reported when adjusted for *MSI* and tumor sidedness. A recent comprehensive retrospective study on the clinical characteristics and genomic features EO- and LO-CRC among large group of patients from Memorial Sloan Kettering Cancer Center (MSKCC) reported no distinctive genetic or molecular differences between early and late onset CRC [62]. The variability in these studies could be explained by the used molecular and genetic tests, the included genes in the study, and the variation in the included cohort study [63].

Despite no difference at the genetic and molecular level, the two cohorts of patients do differ in their clinical parameters and presentation. Majority (80%) of EOCRC patients has left sided tumor and 30% have rectal involvement [62]. EOCRC patients are more likely to present with synchronous and metachronous cancers [64]. CRC at young age has mostly poorly differentiated histopathology with mucinous and signet ring features [65]. EOCRC patients found to present usually at late stage with an aggressive disease and high incidence of cancer recurrence and metastasis [66].

The late and advanced stage at presentation could be very largely due to the reported significant delay in diagnosis and lack of recognition of symptoms [62]. As reported earlier, there is a delay of an average of 6 months from appearance of symptoms till diagnosis and usually they are evaluated by about 3 different physicians till a diagnosis is made. In the other hand, there is no explanation yet for the distinct histopathological features and other reported clinical variations between the two cohorts. This highlights the need for more molecular studies to decipher a potential unknown germline mutation that might provide an explanation for such distinctive clinicopathological picture. It has even proposed that there is a possibility that low risk allels might be responsible for such presentation that worth further studying [63].

### 4.3 Management of EOCRC

According to 2020–2022 cancer facts and figures report, about 37% of CRC patients in the USA are diagnosed at early stages. Approximately, 35% and 21% of CRC patients are diagnosed at stage III and IV respectively. Early stages disease has favorable prognosis with 5-year survival rate of 90% declining to 71% for stage III and 14% for advanced disease [26].

Physicians tend to aggressively treat advanced stage CRC in young patients despite the accumulative evidence of no improvement in survival [[67], [68], [69], [70]]. In a study done at Memorial Sloan Kettering Cancer Center (MSKCC), there was no significant difference in response to the management plan and chemotherapy regimen between EOCRC and LOCRC. They reported that the first line therapy for both groups was luoropyrimidine and oxaliplatin with or without bevacizumab. The reported radiographic response and the median overall survival was comparable between the two groups with not significant statistical difference [62].

Knowing that EOCRC are more prone to develop treatment and disease related long erm side effects, a multidisciplinary and detailed discussion of their management plan is very critical and essential [26]. Fertility is considered a very critical aspect that should be addressed and discussed with patients with EOCRC prior to commencing treatment. The current NCCN guidelines for colon cancer and rectal Cancer does not distinguish between EOCRC and LOCRC and we agree with this approach as there are no evidence to suggest treatment modification for CRC based on the age of onset.

## 5 Survivorship

### 5.1 Survivorship challenges for EOCRC

Despite the reported aggressive nature and advanced stage at diagnosis, the survival of EOCRC patients is found to be equivalent to LOCRC patients if not better [10,[69], [70], [71], [72]]. According to SEERs data, stage specific survival rate is better in those below 50 compared to those >50 years old. But when survival was stratified by age group, those below 35 years old found to have poor prognosis in general [67,73]. This could suggest a unique molecular and biological signature that tend the disease to be aggressive and fatal. In fact, age is considered a worse prognostic factor where the younger the patient, the worse the prognosis [73]. Due to the recent advancement in cancer therapy that has improved the survival rate of these patient over 80%, more young adult patients are surviving cancer [74].

**Fig. 5:**
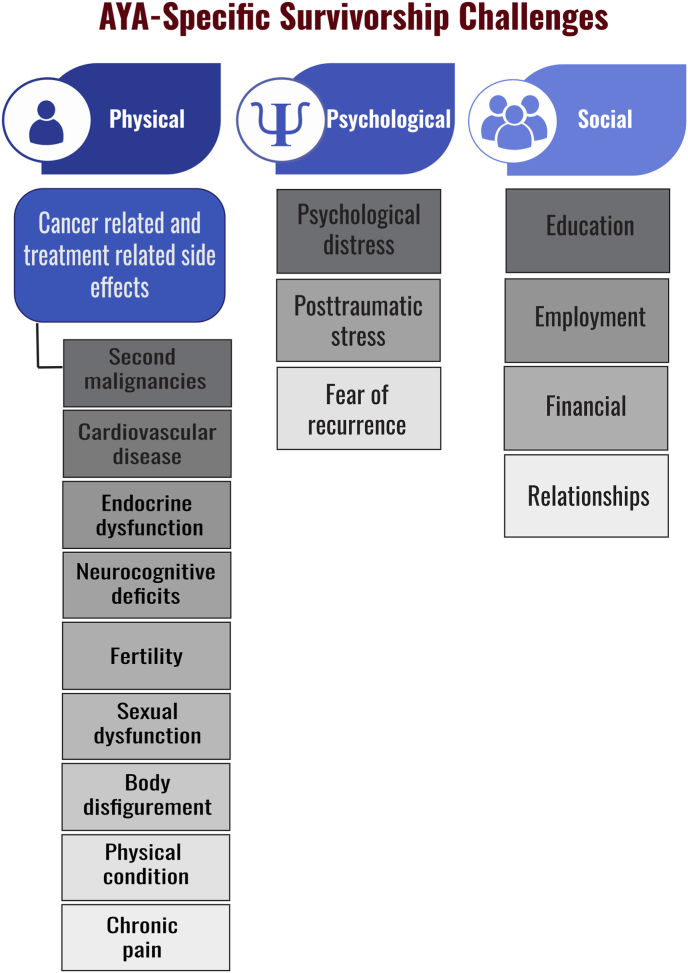
Common challenges perceived by young adult survivors of colorectal cancer.

Young patients who survive CRC face similar challenges faced by older counterpart such as cancer and treatment related long side effects, fear of recurrence and other psychosocial issues (Fig. 5). A recent qualitative study highlighted unique survivorship experiences that young patients pass through across different transitional stages which requires a personalized survivorship plan [75]. Majority of their psychological distress is due to the process of altering their life plan to adjust to the unwelcomed guest. Several physical and psychological symptoms have been reported that are either cancer related or treatment related such as pain, chronic fatigue, anorexia, dyspnea, mental confusion, bowel obstruction, genitourinary problems and others [[2], [3], [4],^60^,^61^]. Some of the underrecognized issues that need to be involved and considered in the management of such patients are the family planning, mental health, job and financial security, psychosocial issues and sexual dysfunction [76]. Unexpectedly, it appears that young adults endure severe symptoms burdens to stay as independent as possible. Therefore, a proactive intervention should be offered to these patients with support that focus on psychosocial spiritual, physical and mental concerns.

In a study that evaluated the long-term symptoms of both young and old survivors over an average period of 10.8 years, young survivors reported higher scores for several psychophysical symptoms compared to older counterpart [77]. Some of the reported symptoms are anxiety, bowel abnormal movements, hair loss and body image embarrassment. Furthermore, young CRC survivors are more likely to report lower social functioning scores which has been found to be largely associated with low educational level, presence of ostomy and pre-existing residual symptoms. This largely impacted the quality of life of CRC survivors [78,79]. Fortunately, 5 years after curative treatment, EOCRC survivors found to have no difference in common cause of death compared to any other person in their age [80]. After 10 years of cancer treatment, secondary cancer is considered the most common cause of death followed by cardiovascular diseases [80].

The short and long term survivorship concerns that EOCRC survivors are facing are not well studied. NCCN has proposed three survivorship models for young patients with cancer. They largely depend on the availability of resources and the burden of the disease. One model is lead by cancer professional in a cancer based care facility. The second model is lead by general practitioner and in a primary care setting. The third is a hybrid that include both professions [81]. More studies are needed in order to decipher the severity and magnitude of early and late survivorship complications and to evaluate these models and their effectiveness in improving the quality of life of young survivors.

### 5.2 Palliative & supportive care care for EOCRC

In younger adults with early-onset CRC, the demand for early introduction of palliative care is vital. Palliative care providers need to be aware of the status of EOCRC and their concerns and they should be available for frequent, interdisciplinary assessments and plan adjustments. The premature perception of death and the feeling of weakness and inability to achieve future life milestones due to the anticipated deterioration and death would aggravate the patients’ psychosocial and spiritual distress [82,83].

**Fig. 6:**
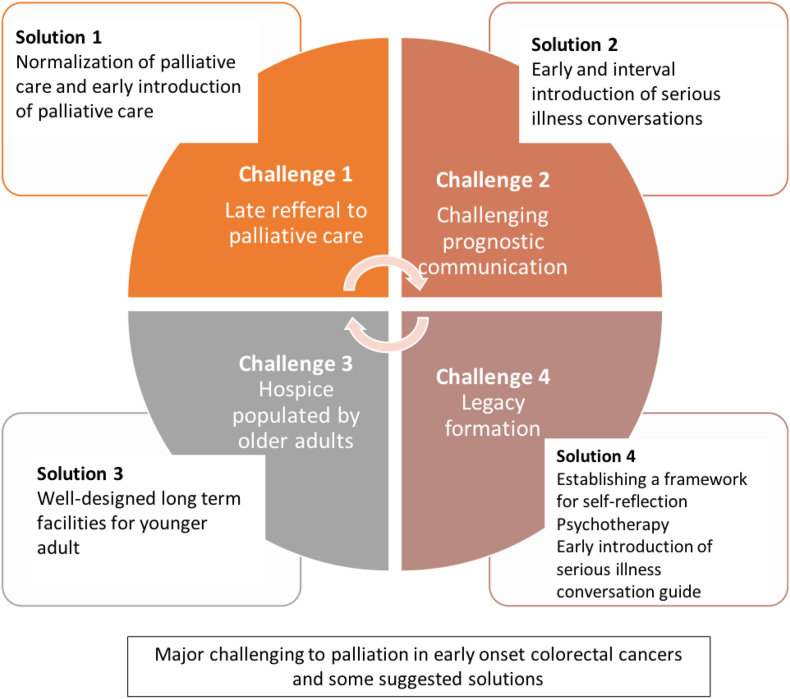
Common challenges in palliative care of young adult patients with colorectal cancers and potential solutions.

Despite recognizing the essential role of palliative care, consultations are made late in disease trajectory. The term ‘‘palliative care’’ may be a barrier to early referral because of the perception of lowering hope and inflicting misery to patients and families. This is in part justified whilst treating physicians find communications round introducing palliative care for more youthful adults. More efforts are needed to impede palliative care at early stage in young cancer patient management plan [[84], [85], [86]]. Communicating disease prognosis with young patients with unexpected serious illness is inherently difficult [[87], [88], [89]]. There is an ongoing efforts to construct both conceptual and practical models to improve such communication (Fig. 6) [90]. Such communication is thought to be introduced early at the time of diagnosis and to be repeated frequently [87,[90], [91], [92]].

Young patients with cancer may want to be remembered perhaps not by the length or importance of their accomplishments but by a unique mark on the world and feel like they made a difference in some unique way. Young cancer patients, especially those with children, may focus on what their children will remember and how not to be forgotten in their eyes [93]. Actively addressing the unique and changing needs of the young cancer patients is essential to gaining insight and establishing a framework for self-reflection. Offering anticipatory guidance, labeling grief, and empathy about developmentally appropriate losses helps the patient feel understood and respected [93].

## 6 Conclusion

It is obvious that young cancer patients in general and EOCRC patients specifically have a distinct environmental, behavioural and likely genetic profiles that necessitate a personalized cancer continuum. Personalized medicine as defined by the National Institutes of Health (NIH) is “an emerging approach for disease treatment and prevention that takes into account individual variability in genes, environment, and lifestyle for each person.“. If such continuum is developed, it will move the cancer care from one size fits all into a targeted individualized continuum starting from personalized screening recommendation to targeted therapy and patients centered survivorship care which is thought to improve outcome, wellbeing and quality of life of young cancer patients.

## Ethical approval

Not Applicable

## Sources of funding

This work was supported by SQU Deanship of research fund and Burjeel Medical City.

## Authors contribution

Adhari AlZaabi: Concept, design, manuscript write up

Amna AlHarrasi: Manuscript write up

Atika AlMusalami: Manuscript write up

Nawal AlMahyijari: Manuscript write up

Khalid Al Hinai: Manuscript write up

Hum**a**id ALAdawi: Manuscript write up

Humaid O. Al-Shamsi: Manuscript write up, Proofread

## Registration of research studies

Name of the registry:

Unique Identifying number or registration ID:

Hyperlink to your specific registration (must be publicly accessible and will be checked):

## Guarantor

Adhari AlZaabi

## Consent

Not Applicable

## Declaration of competing interest

The authors have no conflicts of interest to declare.
